# Synergistic Effect of 4A Molecular Sieve on Intumescent Ternary H-Bonded Complex in Flame-Retarding of Polypropylene

**DOI:** 10.3390/polym15020374

**Published:** 2023-01-10

**Authors:** Qilin Wen, Yinghong Chen, Xin Wang, Haoran Pei

**Affiliations:** State Key Laboratory of Polymer Materials Engineering, Polymer Research Institute of Sichuan University, Chengdu 610065, China

**Keywords:** 4A molecular sieve, synergistic effect, hydrogen-bond complexation, intumescent flame retardant, polypropylene

## Abstract

In this study, a ternary hydrogen (H)-bonded complex intumescent flame retardant (TH-IFR) of melamine (ME) · phosphoric acid (PA)…pentaerythritol (PER) was synthesized through hydrothermal reaction. The combination of the synthesized TH-IFR with 4A molecular sieve as the synergist was used for the first time to improve the flame retardancy of polypropylene (PP). The involved structure, morphology, flame retardancy, flame-retarding mechanism and mechanical properties of the prepared PP composites were systematically investigated. The results show that incorporation of 1 wt% synergist 4A shows the optimum synergistic effect, and the flame retardancy and mechanical properties of the flame-retarded (FR) PP composites are significantly improved. Incorporation of 4A could change the pyrolysis process of the entire system and promote the char-forming chemical interaction, thereby further enhancing the flame retardancy of FR PP composite. The synergistically flame-retarding mechanism of 4A is explained by the significantly improved quality and quantity of the solid-phase char layer, which is formed through generation of SiO_2_ and Al_2_O_3_ substances, and also participation of PP macromolecular chains in the final char layer formation during burning. Furthermore, the improved dispersion and compatibility of TH-IFR in the composite is largely beneficial to the improvement of flame retardancy and mechanical properties.

## 1. Introduction

Polypropylene (PP), as one of the important five general purpose plastics with excellent mechanical properties, chemical resistance, easy moldability and non-toxicity, and it is mainly used in construction, transportation and electronics [1,2]. However, its inherent flammability limits its application in many fields with fire-proof requirements [3]. Furthermore, PP is easy to cause secondary fire due to its molten droplets and will produce a large amount of toxic smoke during the combustion process [4,5,6]. These shortcomings would cause huge potential danger to the safety of human life and property. Therefore, it is necessary to improve the flame retardancy of PP by incorporating flame-retardant additives.

Recently, with the increasing attraction to health and environmental issues, people have turned their attention to the development and use of environmentally friendly and highly-efficient intumescent flame retardants (IFR) [7,8,9,10]. The IFR system generally contains an acid source, carbon source and gas source. Due to the poor compatibility of the mixed-flame retardants with the matrix, it is easy to deteriorate the properties of material [11,12]. Comparatively, the ternary integrated intumescent flame retardants (TIRs, with acid source, gas source and char former in one flame-retardant molecule) have lower polarity and better compatibility with the PP matrix [13,14,15]. Furthermore, the TIRs-added PP composites have reduced moisture absorption, and the incorporated TIRs are less prone to exudation [16,17].

Based on the advantages of ternary integrated intumescent flame retardant (TIR), many efforts have been made to improve the flame retardancy of PP. For example, Zhu et al. [18] synthesized a novel TIR hyper-branched triazine-piperazine pyrophosphate (HTPPP) by using cyanuric chloride, 1-boc-piperazine and phosphoric acid. They found that when HTPPP alone was loaded at 25 wt%, the limiting oxygen index (LOI) value of PP/HTPPP composite reached 30.5% and achieved V-0 rating in vertical burning tests (UL-94). Qi et al. [19] synthesized a novel TIR poly-(spirocyclic pentaerythritol bisphosphonate-1,3,5-triazine-O-bicyclic pentaerythritol phosphate) (PSTBP). The results showed that the LOI value of PP/PSTBP composite reached 30.5% and achieved V-0 rating in UL-94 test when the PSTBP was added at 30.0 wt%. Compared with PP/APP/PER composite, the PP/PSTBP composite showed a better water resistance. Zheng et al. [20] synthesized a mono-component, cellulose-based and intumescent halogen-free flame retardant (HECPM) by introducing IFR groups in the structure of cellulose. The results showed that there is a synergistic flame-retardant effect between HECPM and expandable graphite (EG). When HECPM/EG (1/3) was added at 30 wt%, the LOI value and UL-94 vertical burning rating of the prepared PP composite could reach 31.5% and V-0, respectively. Liu et al. [21] synthesized a mono-component flame-retardant additive of poly (dimethylol melamine piperazine pyrophosphate) (PDMPP) by using formaldehyde, melamine and piperazine pyrophosphate. It was found that the prepared PP/23 wt% PDMPP composites could either achieve a V-0 rating of UL-94 before and after water resistance tests, and the LOI value decreased slightly from 26.7% to 26.3%.

As a matter of fact, the addition of TIRs alone could show their higher efficiency only at a high loading [22]. As we know, incorporation of a suitable synergist is effective [23]. Many studies have achieved the desired flame-retardant effect by adding different inorganic synergists, including graphene, montmorillonite, metal oxides, zeolite and polyoxometalate [24,25,26,27,28]. The 4A molecular sieve (4A) is a synthetic aluminosilicate with an effective aperture of about 0.4 nm and a three-dimensional network structure composed of SiO_4_ and AlO_4_ tetrahedrons combined with the common oxygen atoms [29,30,31]. Its unique structure and composition make 4A have good thermal stability, absorptivity, ion exchangeability and catalytic property. Recently, various types of molecular sieves have been used alone as flame retardants to achieve a certain flame-retardant effect, or used as synergists to achieve a good synergistic flame-retardant effect [30,31,32,33]. Thus, 4A could be a promising candidate as a flame-retardant synergist in PP flame retardancy.

In this study, a novel ternary hydrogen (H)-bonded complex intumescent flame retardant (TH-IFR) of melamine (ME) phosphoric acid (PA)·pentaerythritol (PER) was synthesized through a moderate and environmentally friendly hydrothermal reaction. Then, the synthesized TH-IFR and the flame-retardant synergist 4A were combined to improve the fire safety of the flame-retarded (FR) PP materials. The structure, morphology, flame retardancy, thermal performance and mechanical property of the prepared FR PP composites are systematically investigated. Additionally, the related flame-retarding mechanism is carefully discussed.

## 2. Experimental

### 2.1. Materials

Phosphoric acid (PA, 85%) and melamine (ME) were all analytically pure and purchased from Kelong Chemical Reagent Corporation (Qionglai, Sichuan, China). Pentaerythritol (PER) was analytically pure and purchased from Tianjin Guangfu Fine Chemical Research Institute (Tianjin, China). The 4A molecular sieve (4A) powder with an effective aperture of about 0.4 nm was commercial grade and supplied by Jiangxi Xintao Technology Co., Ltd. (Pingxiang, Jiangxi, China). Polypropylene (PP, a homopolymer named T30S, melt flow rate = 3.4 g/10 min at 230 °C) was purchased from Dushanzi Petrochemical corporation (Kelamayi, Xinjiang, China).

### 2.2. Synthesis of TH-IFR

**Figure 1:**
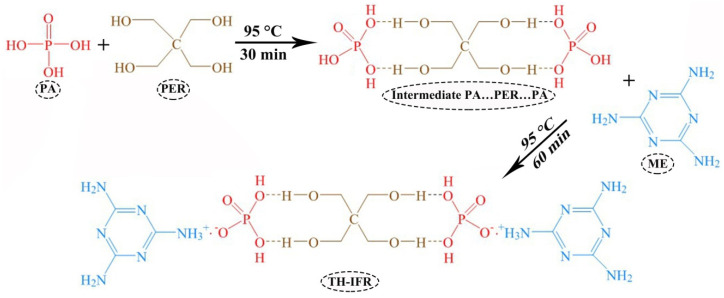
The schematic diagram for synthesizing TH-IFR.

The TH-IFR was synthesized through a hydrothermal reaction [34], as shown in Figure 1. First, 2.0 mol phosphoric acids were dropped into the deionized water in a three-necked flask with mechanical stirring for 10 min at 95 °C. After that, 1.0 mol pentaerythritols were added and stirred for 30 min at 95 °C. Finally, 2.0 mol melamines were added slowly and stirred vigorously for the additional reaction time of 60 min at 95 °C. The finally obtained reaction mixture was filtered and further dried at 120 °C for 24 h. The dried flame-retardant products were effectively pulverized using a multi-functional pulverizer. The obtained flame-retardant powders were sieved into 80 mesh particles for preparation of the flame-retarded PP materials.

### 2.3. Preparation of Flame-Retarded PP Composites

The synthesized TH-IFR, 4A and pure PP were homogeneously mixed in a high-speed mixer, according to the formulation shown in Table 1. The well-mixed ingredients were extruded in a co-rotating twin-screw extruder (Φ: 25 mm, L/D: 44:1, TSSJ25/44, Angemei Company, Chengdu, China) with a screw speed of 200 rpm and at a temperature of 180 °C. The water-cooled extrudates were cut into pellets and dried at 80 °C for 8 h. The dried pellets were injection molded in an injection machine (MA 1200-SMS-A, Haitian Group Company, Ningbo, China) at 200 °C to obtain standard combustion and mechanical test samples (Figure S1).

### 2.4. Characterization

The Fourier transform infrared (FT-IR) spectra of samples were obtained by using a FTIR spectrometer (Nicolet 6700, Thermo Fisher Scientific, Waltham, MA, USA) at a range of 4000–500 cm^−1^. For the liquid intermediate PA…PER…PA, before measurement, it was coated on a salt tablet priorly prepared.

The composite samples were fractured by immersion in liquid nitrogen. Then, the fracture surface morphology of composite samples were observed by field emission scanning electron microscopy (FESEM, Apreo S HiVoc, FEI, Hillsboro, OR, USA) with an accelerating voltage of 5 kV. The additives and carbon residues were also observed by FESEM and analyzed using an energy dispersive X-ray spectrometer (EDS, Octane Elect Super, EDAX, Philadelphia, PA, USA) equipped with the above FESEM.

The limiting oxygen index (LOI) of samples were estimated using an oxygen index meter (JF-3, Nanjing, China) with a dimension 120 × 10 × 4 mm^3^, according to ASTM D2863. The UL 94 vertical burning tests of samples were performed using a horizontal vertical flame tester (JT-HVR5450, Dongguang, China) with a dimension 125 × 13 × 3.2 mm^3^, according to ASTM D3801. Before testing, the samples were well conditioned at a room temperature for 48 h. The cone calorimeter test (CCT) of samples was performed using a cone calorimeter (iCone, FTT, East Grinstead, West Sussex, UK) with a dimension of 100 × 100 × 3 mm^3^, according to ISO 5660-1, at a heat flux of 50 kW/m^2^. After the CCT test, the height of the left charred layers was carefully measured [22].

The thermal stability of samples was determined on a thermogravimetric analyzer (TGA, Q50, TA Instruments, Newcastle, DE, USA) at a heating rate of 10 °C/min from 30 °C to 700 °C under N_2_ atmosphere (flow rate of 60 mL/min). The crystallization and melting behaviors of the samples were investigated by differential scanning calorimetry (DSC; Q20, TA Instruments, Newcastle, DE, USA) at a heating rate of 10 °C/min from 30 °C to 220 °C under N_2_ atmosphere (flow rate of 50 mL/min).

The Raman spectra of carbon residue were recorded by a Raman spectrometer (HR Evolution, HORIBA, Paris, France) equipped with the excitation of a 532 nm laser.

The tensile properties of samples were measured by using a universal testing machine (DWD-10KN, Sichuan DEXKCYQ Instrument Company, Chengdu, China), according to ASTM D638, with a crosshead speed of 50 mm∙min^−1^. The five specimens were measured for each FR PP composition sample and the average value was used.

## 3. Results and Discussion

### 3.1. Synthesis and Characterization of TH-IFR

**Figure 2:**
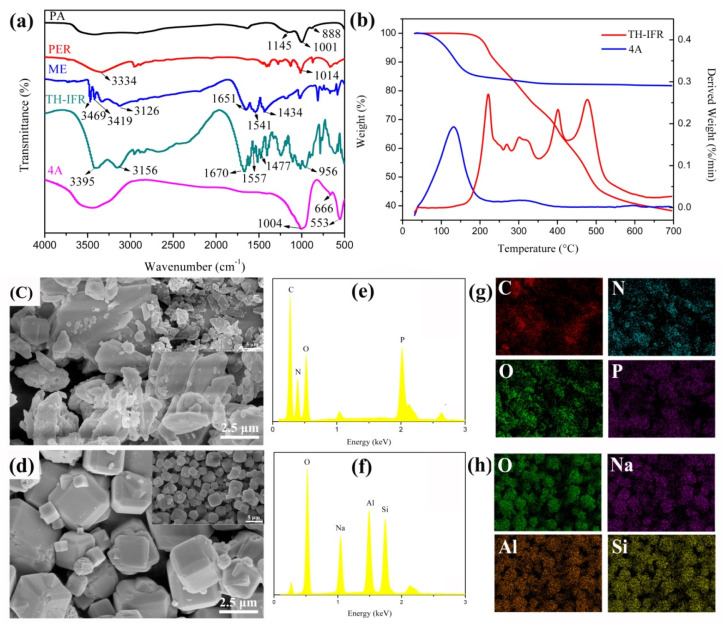
FT-IR spectra of PA, PER, ME, TH-IFR and 4A (**a**); TGA curves of TH-IFR and 4A (**b**); FESEM images of TH-IFR (**c**) and 4A (**d**); EDS spectra of TH-IFR (**e**) and 4A (**f**); elemental mapping images of TH-IFR (**g**) and 4A (**h**).

In this study, an environmentally friendly hydrothermal reaction synthesis strategy was adopted to prepare the ternary H-bonded complex intumescent flame retardant (TH-IFR) from melamine (ME), phosphoric acid (PA) and pentaerythritol (PER). In the first step, the PA was reacted with PER to form intermediate PA·PER·PA through H-bonded complexation reaction; in the second step, the formed intermediate PA…PER…PA was used to react with ME to synthesize the final product TH-IFR. Figure 2 shows the FT-IR spectrum of the synthesized TH-IFR. For convenience of comparison, the FT-IR spectra of all reactants (PA, PER and ME) and the synergist 4A were provided in Figure 2. In addition, the FT-IR spectrum of the intermediate PA…PER…PA was shown in Figure S2. As can be seen, there are the typical characteristic peaks of the reactant PA (3419 cm^−1^ for P-OH bond, 1145 cm^−1^ for P=O bond, 1001 and 888 cm^−1^ for P-O bond), PER (3334 and 1014 cm^−1^ for C-OH bond) and ME (3469, 3419, 3126 and 1651 cm^−1^ for N-H bond, 1541 and 1434 cm^−1^ for C=N bond) appearing [12]. Compared with the above-mentioned reactants of PA (3419 cm^−1^) and PER (3334 cm^−1^), the characteristic peak (3389 cm^−1^) of the –OH in intermediate PA·PER·PA shifts to lower (from 3419 to 3389 cm^−1^, i.e., the red shift) and higher (from 3334 to 3389 cm^−1^, i.e., the blue shift) wavenumber direction, respectively. This means that the new intermolecular H-bonds have been formed between PA and PER [34,35]. The new emerging characteristic peak of TH-IFR at 3395 and 3156 cm^−1^ and the changes in characteristic peaks (from 1651 to 1670 cm^−1^, 1541 to 1557 cm^−1^ and 1434 to 1477 cm^−1^) were all assigned to the salt reaction between intermediate PA·PER·PA and ME [5,36]. All the above results demonstrate that the TH-IFR was successfully synthesized. For the spectrum of 4A, the characteristic peak at 1004 cm^−1^ and 666 cm^−1^ was assigned to Si-O-Si stretching vibration. The characteristic peak at 553 cm^−1^ was assigned to Si-O and Si-O-Al stretching vibration [15].

The TGA curves of TH-IFR and synergist 4A are shown in Figure 2. It can be seen that the decomposition of TH-IFR is divided into four stages (Figure 2b). The first stage is in the range of 150~258 °C, which could be attributed to water loss and degradation of the incompletely reacted PER [37]; the second stage is in the range of 280~350 °C, which could be caused by the release of water and ammonia due to the existence of hydroxyl and amine groups; the three stage is in the range of 350~427 °C, which is attributed to the formation of char layer; the four stage falls in the range of 427~650 °C, which could be ascribed to the further degradation of the char layer and the formation of the final intumescent carbon layer. Moreover, the char yield of TH-IFR at 700 °C is 38.29%, revealing a good char-forming capability. As for the synergist 4A, there is only one degradation process occurring in the range of 50~150 °C, which could be attributed to the adsorbed water loss, and the char yield is as high as 81.78% at 700 °C, showing its excellent high-temperature thermal stability.

The FESEM and EDS characterization tools were further used to confirm the structure and composition of TH-IFR and 4A. The results are shown in Figure 2. It can be seen that the TH-IFR exhibits a rough surface and an irregular blocky structure, and its particle size ranges in 0.5-20 µm (Figure 2c). The 4A exhibits a relatively smooth surface and a regular blocky structure, and its particle size ranges in 2-4 µm (Figure 2d). The elemental composition of TH-IFR mainly consists of C (36.26%), N (30.47%), O (26.73) and P (6.54%) (Figure 2e and Table S1). The elemental mapping images demonstrate a uniform dispersion of these elements (Figure 2g), indicating that the TH-IFR was successfully synthesized. In addition, the elemental composition of 4A is mainly composed of O (49.83%), Na (13.41%), Al (18.02%) and Si (18.74%) (Figure 2f and Table S1).

### 3.2. Characterization of Flame-Retarded PP Composities

#### 3.2.1. Morphology Analysis

**Figure 3:**
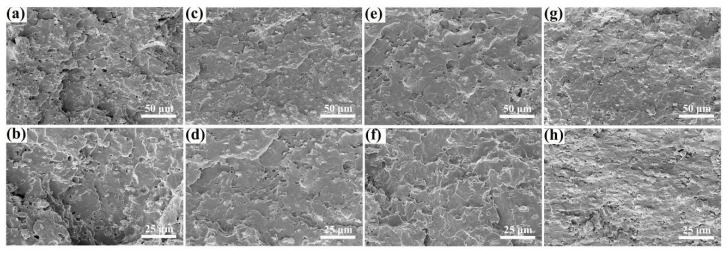
The fractured surface FESEM images of TH-IFR/PP (**a**,**b**), TH-IFR/4A/PP1 (**c**,**d**), TH-IFR/4A/PP2 (**e**,**f**) and TH-IFR/4A/PP4 (**g**,**h**) samples.

The fractured surface morphologies of TH-IFR/PP, TH-IFR/4A/PP1, TH-IFR/4A/PP2 and TH-IFR/4A/PP4 were observed, as shown in Figure 3. It can be seen that the fractured surface of TH-IFR/PP is relatively rough and some holes can be observed (Figure 3a,b). Comparatively, after adding 4A, the fractured surface of TH-IFR/4A/PP1 becomes smoother and the number of holes significantly decreases (Figure 3c–f), indicating that the dispersion and compatibility were improved to a certain degree. The reason for this may be that the adsorption property of 4A particles is beneficial to the improvement in dispersion and compatibility of TH-IFR in matrix [29]. Furthermore, with the further increase of 4A loading, the fractured surface of TH-IFR/4A/PP4 becomes rough again, and some aggregated particles appear (Figure 3g,h). The above morphology results would obviously be beneficial to the enhancement in the flame retardancy and mechanical properties of the 4A incorporating FR PP composites.

#### 3.2.2. Flammability Performance

The LOIs and UL-94 ratings of pure PP and FR PP composites are shown in Table 1. It can be seen that the LOI of pure PP is 19.0% and fails to pass the UL-94 rating. With the introduction of different compositions of TH-IFR and 4A molecular sieve, the LOI of the FR PP composites rises first and then decreases. Although the LOI of the TH-IFR/PP FR material is enhanced to 27.6%, this material only passes the UL-94 V-1 rating. Comparatively, the LOI of TH-IFR/4A/PP1 (with 1.0 wt% 4A) has the highest value of 30.1% and passes the UL-94 V-0 rating. However, with further increasing the 4A content to 4.0 wt%, the LOI of TH-IFR/4A/PP4 continuously decreased to 25.9% and fails to pass the UL-94 rating. These results show that there is a remarkable synergism between the main flame retardant of TH-IFR and the 4A molecular sieve (e.g., adding 1 wt% synergist 4A achieves the best flame retardancy). This is possibly because the incorporated 4A as a catalyst facilitates formation of the expanded and stable carbonaceous residues [38,39,40] and, hence, helps to inhibit the propagation of fire as a physical barrier, resulting in better flame retardancy of the composites. However, when the 4A content increases to higher than 1.0 wt%, on one hand, at the same flame retardant loading, the increase of 4A loading means the decrease in the relative content of the working flame retardant; on the other hand, the 4A molecular sieves possess the large specific surface area and, hence, the great adsorbability. Hence, the higher loading of 4A molecular sieves (e.g., ≥2.0 wt%) would easily lead to the agglomeration of flame retardants (Figure 3g,h). The above factors would of course cause the decrease in flame retardancy (e.g., LOI) of FR PP material. That is to say, there is no such synergistic effect in flame retardancy at a higher flame retardant loading (e.g., 2.0 wt%).

**Figure 4:**
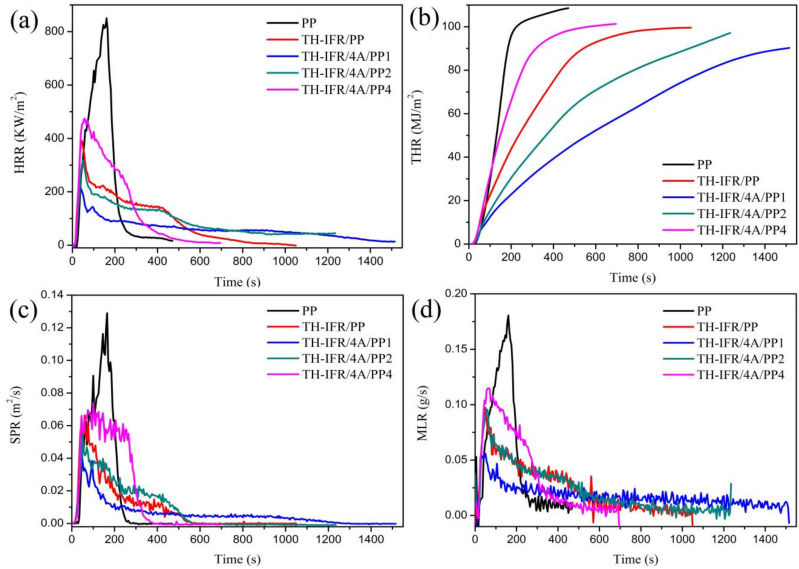
The HRR (**a**), THR (**b**), SPR (**c**) and MLR (**d**) curves of pure PP, TH-IFR/PP, TH-IFR/4A/PP1, TH-IFR/4A/PP2 and TH-IFR/4A/PP4 FR material.

The cone calorimeter test (CCT) proves to be an effective way to simulate real fire hazards. The heat release rate (HRR), total heat release (THR), smoke production release (SPR) and mass loss rate (MLR) curves of the pure PP, TH-IFR/PP and TH-IFR/4A/PP1 samples are shown in Figure 4. The corresponding parameters are listed in Table 2. The pure PP exhibits a longer time to ignition (TTI) of 28 s and burns violently with a pHRR value of 850.19 kW/m^2^. The TTI of FR PP composites (21~23 s) is lower than that of pure PP, which should be attributed to the change in the decomposition process of PP composites [41]. Comparatively, the pHRR and THR values of the TH-IFR/4A/PP1 FR sample are further reduced (850.19 to 212.42 kW/m^2^ and 108.52 to 90.04 MJ/m^2^). This result again indicates existence of the synergistic flame-retardant effect between TH-IFR and 4A. Obviously, the peak smoke production release (pSPR), peak mass loss rate (pMLR) and average effective combustion (av-EHC) follow the following order of PP > TH-IFR/4A/PP4 > TH-IFR/PP > TH-IFR/4A/PP2 > TH-IFR/4A/PP1. In addition, the fire performance index (FPI, the ratio of TTI to pHRR) and the char residues are in reverse order. The above results indicate that the incorporation of TH-IFR fillers could improve the flame retardancy of the composite and reduce release of the amount of flammable gases through combining the gas-phase (producing non-flammable gases such as H_2_O, NH_3_ and CO_2_) and solid-phase (producing stable and intumescent carbon layers) flame-retardant effects. However, the 4A could further strengthen the effects mainly through a solid-phase flame-retardant effect (catalytic formation of more stable carbon layers) [38,39,40]. As a result, incorporating 1 wt% 4A can exert the optimal synergistic flame-retardant effect and significantly improve the flame retardancy of the FR PP system.

#### 3.2.3. Thermal Performance

**Figure 5:**
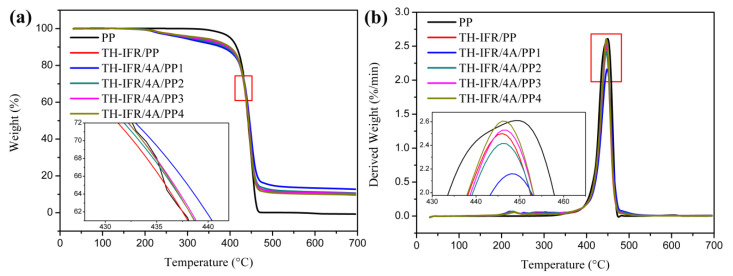
The TG (**a**) and DTG (**b**) curves of pure PP and FR PP composites.

The thermal stabilities of these samples under N_2_ atmosphere were characterized using TGA, as shown in Figure 5 and Table 3. The evaluation parameters include the onset decomposition temperature (T_onset_, 5% mass loss temperature), the temperature at the maximum decomposition rate (T_max_), the maximum decomposition rate (MDR), the real char yield at 700 °C (C_700 °C_) and the theoretical char yield at 700 °C (T_700 °C_). The variation degree of char yield between the real value and the theoretical value is calculated by the following equation:

(1) $$\mathrm{Variation}=\frac{C_{700\;{}^{\circ}C}-T_{700\;{}^{\circ}C}}{T_{700\;{}^{\circ}C}}\times 100\%$$

It can be seen that in the whole degradation process, pure PP begins to decompose at 400 °C, and only one T_max_ occurs at 449 °C, which corresponds to the MDR of 2.61 %/min, and there is no char residue remaining at 700 °C. This indicates that pure PP easily burns completely. Compared with pure PP, the T_onset_ of the FR PP composites are obviously lower. This is because the incorporated TH-IFR additives decompose earlier and show a positive influence on the degradation and carbonization of the composites. The reason for the lowest T_onset_ (290 °C) of TH-IFR/4A/PP1 FR material is that the introduction of the 4A synergist would change the pyrolysis process of the system [31], resulting in earlier degradation and carbonization of the entire system. In the meantime, the incorporation of the excessive 4A particles (≥2 wt%) would delay the degradation and carbonization of the FR composites. The T_max_ of the composites is lower than that of pure PP, and this may be due to the decomposition of the flame retardants for release of acid sources at lower temperature [42]. The value of MDR presents a first decreasing and then increasing tendency. The reduction of MDR is beneficial to the char formation of PP composites, thus increasing the char yield. Obviously, except for the TH-IFR/4A/PP4 sample, the C_700 °C_ of the other composites is higher than T_700 °C_, indicating the presence of the chemical interaction among the flame retardant, synergist and PP matrix, and participation of PP macromolecular chains in the carbon formation. The variation degree of char yield could be used to describe the strength of the chemical interaction. The higher the variation degree is, the stronger the char-forming chemical interaction of the intumescent system, leading to the better flame retardancy of the FR PP composites. The TH-IFR/4A/PP1 sample exhibits the highest variation degree of 43.39%, suggesting that adding 1 wt% 4A could achieve the best synergistic effect with TH-IFR. In particular, the TH-IFR/4A/PP4 exhibits a negative variation degree, and this may be because of the relatively lower loading of TH-IFR and/or the possible agglomeration of 4A particles. Furthermore, it is worth noting that the change regularity of the C_700 °C_ of the samples is consistent with that of the LOI and UL-94 test results.

**Figure 6:**
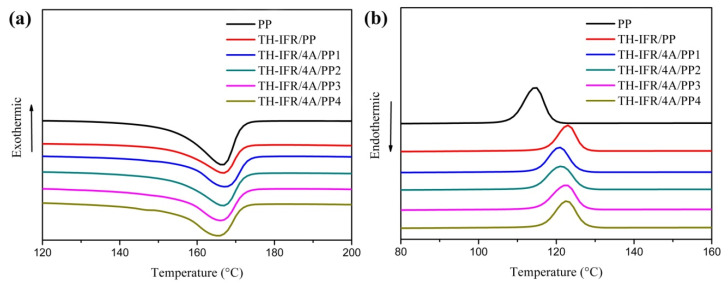
The DSC melting (**a**) and crystallization (**b**) curves of pure PP and FR PP composites.

The DSC melting and crystallization curves of samples are shown in Figure 6, and the corresponding parameters are summarized in Table 4, including the crystallization temperature (T_c_), the crystallization enthalpy (ΔH_c_), the melting temperature (T_m_), the melting enthalpy (ΔH_m_) and the crystallinity (X_c_). The X_c_ is calculated according to the following equation:

(2) $$X_{c}=\frac{\Delta H_{m}}{f\Delta H_{0}}$$

where ΔH_m_ is the melting enthalpy, f is the weight fraction of PP in the composite and ΔH_0_ = 209 J/g is the theoretical melting enthalpy of the 100% crystalline PP [37]. It can be seen that the T_c_ of all the FR PP composites is higher than that of pure PP, indicating that the incorporated TH-IFR fillers and TH-IFR/4A composite fillers both show the heterogeneous nucleation effects on the crystallization of PP macromolecular chains. The value of T_m_ presents a first increasing and then decreasing tendency with increasing 4A content. The first increasing tendency could be attributed to the introduction of the low loading of 4A, which is more favorable for the arrangement of PP macromolecular chains and, hence, the heterogeneous nucleation. The subsequently decreasing tendency could be attributed to the possible agglomeration caused by the adsorbability and large specific surface area of the higher loading of 4A particles (≥2 wt%) [29]. With increasing 4A loading, the PP X_c_ value presents a first decreasing and then increasing tendency. This indicates that the incorporated 4A particles can improve the crystallization of PP to a certain degree (the reason for this could be explained by the heterogeneous nucleation effect of the incorporated 4A particles).

#### 3.2.4. Carbon Residue Analysis

**Figure 7:**
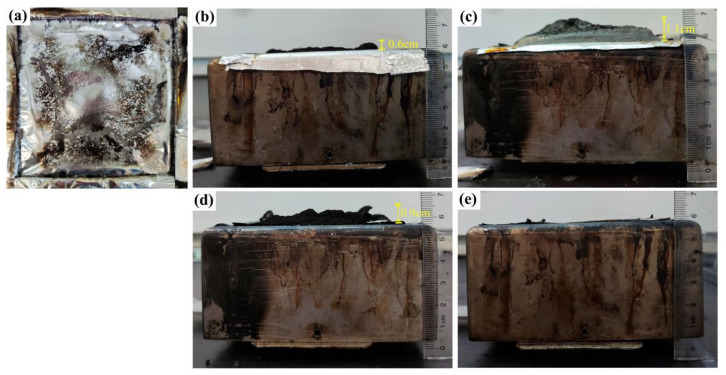
The digital photos of carbon residue for pure PP (**a**), TH-IFR/PP (**b**), TH-IFR/4A/PP1 (**c**), TH-IFR/4A/PP2 (**d**) and TH-IFR/4A/PP4 (**e**).

As for IFR systems, a compact, continuous, stable and intumescent char layer could act as an effective barrier to protect the underlying polymer matrix [43]. The digital photos of the intumescent carbon layers of pure PP, TH-IFR/PP, TH-IFR/4A/PP1, TH-IFR/4A/PP2 and TH-IFR/4A/PP4 samples after CCT are shown in Figure 7 and Figure S3. There is no carbon residue remaining for pure PP. The TH-IFR/PP, TH-IFR/4A/PP1 and TH-IFR/4A/PP2 FR materials show the intumescent char layer formed. The TH-IFR/4A/PP4 FR system show the incomplete char layer formed (Figure 7e and Figure S3). Comparatively, the height of the TH-IFR/4A/PP1 char layers is higher than that of the other FR PP samples, again indicating that the incorporation of 1 wt% 4A has the optimal synergistic effect on carbon formation [38,39,40]. This is the reason why the flame retardancy of the composites could become better after incorporating 4A.

**Figure 8:**
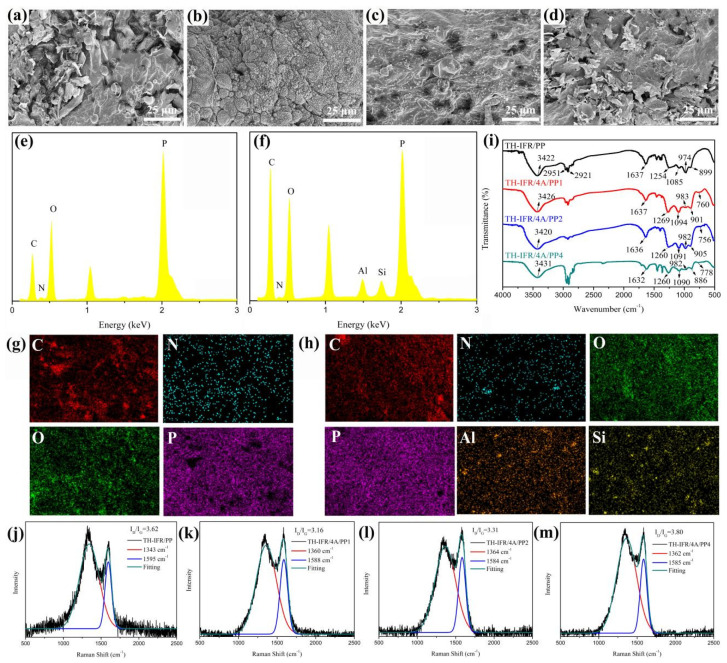
The FESEM images of carbon residue for TH-IFR/PP (**a**), TH-IFR/4A/PP1 (**b**), TH-IFR/4A/PP2 (**c**) and TH-IFR/4A/PP4 (**d**); EDS spectra of carbon residue for TH-IFR/PP (**e**) and TH-IFR/4A/PP1 (**f**); elemental mapping images of carbon residue for TH-IFR/PP (**g**) and TH-IFR/4A/PP1 (**h**); FT-IR spectra of carbon residues for TH-IFR/PP, TH-IFR/4A/PP1, TH-IFR/4A/PP2 and TH-IFR/4A/PP4 sample after CCT (**i**); Raman spectra of carbon residue for TH-IFR/PP (**j**), TH-IFR/4A/PP1 (**k**), TH-IFR/4A/PP2 (**l**) and TH-IFR/4A/PP4 (**m**).

In order to further investigate the synergistic effect of 4A in a FR PP system, the morphology observation of the remaining carbon residues of TH-IFR/PP, TH-IFR/4A/PP1, TH-IFR/4A/PP2 and TH-IFR/4A/PP4 samples and determination of the part element composition after CCT were performed using SEM and the attached EDS, respectively. The results are shown in Figure 8 and Table S2. As can be seen, in the TH-IFR/PP and TH-IFR/4A/PP4 FR samples, there are the carbon layers with many holes and defects formed after combustion (Figure 8a,d), and the structure cannot ensure the good barrier property of the formed carbon layers to the heat and oxygen. On the contrary, the TH-IFR/4A/PP1 FR sample shows the well compact and continuous carbon layers after combustion (Figure 8b), which could provide a good physical barrier effect in preventing the burning of the underlying matrix resin. Furthermore, although the carbon layers of the TH-IFR/4A/PP2 FR sample are relatively compact and continuous, there are an increasing number of holes formed (Figure 8c).

The EDS results show that the carbon residue of TH-IFR/PP sample is mainly composed of the elements C (40.65%), N (3.31%), O (35.17%) and P (20.87%) (Figure 8e and Table S2). However, the carbon residue of the TH-IFR/4A/PP1 sample is mainly composed of the elements C (53.68%), N (1.45%), O (29.71%), P (12.87%), Al (1.20%) and Si (1.09%) (Figure 8f and Table S2). Relative to TH-IFR/PP, the C element content of the 4A-incorporated sample (TH-IFR/4A/PP1) remarkably increases (from 40.65% to 53.68%), confirming that the addition of 4A particles could promote the participation of PP macromolecular chains in the formation of the final char layer. In addition, according to the EDS results, it could be reasonably believed that on the surface of the residual carbon layers, there are the possible SiO_2_ and Al_2_O_3_ particles formed through oxidation of 4A during burning. The above SiO_2_ and Al_2_O_3_ particles could effectively strengthen the carbon layers and, hence, prevent the further oxidative degradation of the carbon layers, which is beneficial to inhibiting heat and gas transfer during the combustion process. Moreover, the element mapping images (Figure 8g,h) demonstrate a uniform dispersion of Al and Si elements in carbon layers (Figure 8h), presenting formation of the good char layers with improved quality in the TH-IFR/4A/PP1 sample. As a result, the incorporated 4A particles improve the flame retardancy of the FR PP composites mainly through promoting the formation of a more thermally stable char layer in the condensed phase.

The FT-IR spectra of the intumescent carbon residues of TH-IFR/PP, TH-IFR/4A/PP1, TH-IFR/4A/PP2 and TH-IFR/4A/PP4 samples after CCT are shown in Figure 8. It can be seen that in the char layers of the TH-IFR/PP FR sample, there are the characteristic peaks of some groups occurring, including N-H bond (3422 cm^−1^), C-H bond (2951 and 2921 cm^−1^), C=C bond (1637 cm^−1^), P=O bond (1254 cm^−1^), P-O-C structure (1085 cm^−1^) and P-O-P structure (974 and 899 cm^−1^) [44,45]. Compared with the TH-IFR/PP FR sample (without 4A molecular sieve), the peak intensities of some functional groups (N-H, P=O, P-O-C and P-O-P) of TH-IFR/4A/PP FR materials (with 4A molecular sieve) are significantly strengthened. In addition, there is also a new peak appearing at 760 cm^−1^, which is caused by the existence of the Al-O-P structure [15] in the char residues. The above results clearly indicate that the incorporated 4A molecular sieves can really effectively catalyze the esterification reaction in the char formation of TH-IFR, thus improving the barrier effect of the intumescent char layers in the system. This is also the reason why the flame retardancy of the composite could be improved after incorporating 1.0 wt% 4A particles. However, it is also noticed that, with further increasing 4A content in 4.0 w%, the intensity of the characteristic peaks of the functional groups above mentioned (particularly C=C, P=O and P-O-C) in the char layer show a decreasing tendency, indicating that the capability for catalyzing char formation weakens. This could explain the decrease in flame retardancy of FR PP materials with increasing the 4A content in the range of over 1.0 wt%.

Raman spectroscopy is also used to evaluate the graphitization degree of carbon residue. The higher graphitization degree of the carbon residue generally means the higher the thermal oxidation resistance, and, hence, the higher flame retardancy of the polymer [46]. Figure 8 shows the raman spectra of the carbon residue of TH-IFR/PP, TH-IFR/4A/PP1, TH-IFR/4A/PP2 and TH-IFR/4A/PP4 samples after CCT. In general, the integrated intensity ratio of band D to G (I_D_/I_G_) could be used to evaluate the graphitization degree of carbon residue. The lower value of I_D_/I_G_ means the higher graphitization degree, indicating that the carbon residue is more stable. As can be seen, the I_D_/I_G_ ratio of the TH-IFR/4A/PP1 (3.16) is lower than that of the other FR PP samples, illustrating that the addition of a small amount of 4A particles is helpful for increasing the graphitization degree. As a result, the more stable char layers could act more effectively as the physical barrier to inhibit the transfer of heat and volatile components during combustion.

#### 3.2.5. Synergistically Flame-Retarding Mechanism

**Figure 9:**
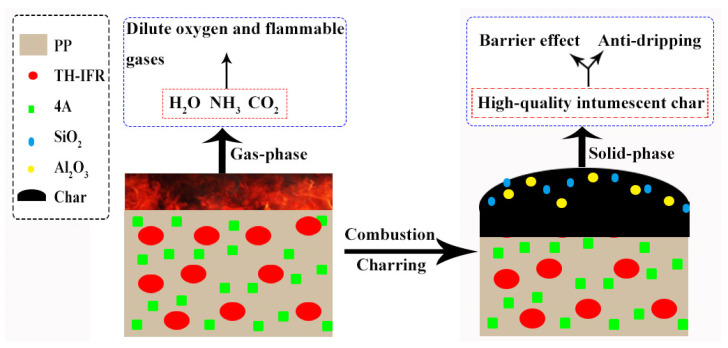
The schematic diagram of the synergistically flame-retarding mechanism.

Based on the above results, the involved flame-retarding mechanism can be illustrated in Figure 9. The synthesized TH-IFR mainly exerts the flame-retardant effects in both gas phase and solid phase. On one hand, during burning, the oxidative decomposition of TH-IFR could generate non-flammable gases, such as H_2_O, NH_3_ and CO_2_, which can reduce heat generation and dilute the external oxygen and flammable gas from the underlying matrix (a gas-phase flame-retardant effect). On the other hand, based on the chemical reactions in TH-IFR, the release of non-flammable gases, as well as the catalytic carbonization of 4A, can ensure the formation of the compact, continuous, stable and intumescent char layers, which can inhibit the transfer of heat and volatile components between the polymer degradation region and the combustion region (a solid-phase flame-retardant effect). As a result, the high-quality intumescent char layers covering the surface of the composite could act as a good physical barrier to effectively protect the underlying substrate. The synergistic effect of 4A plays a dominant role in the solid-phase flame-retardant effect. During the combustion process, on one hand, the incorporated 4A synergists can catalytically promote the participation of PP macromolecular chains in formation of the final char layers, and on the other hand, the 4A synergists could also produce the SiO_2_ and Al_2_O_3_ particles, which have a reinforcing effect on the formed charred layers. All the above effects could help to significantly improve the quality and quantity of the char layers, imparting better flame retardancy to the FR PP composite.

#### 3.2.6. Mechanical Properties

The tensile mechanical properties of pure PP and various FR PP composites were investigated. The corresponding stress-strain curves and the averaged tensile strength and elongation at break are shown in Figure S4 and Table 5, respectively. As can be seen, pure PP has the highest tensile strength (30.3 MPa) and elongation at break (77.5%). It is evident that the mechanical properties of the FR PP composites are negatively affected by the addition of TH-IFR and 4A to some extent. With incorporation of the TH-IFR and 4A fillers, both the tensile strength and elongation at break of FR PP composites show a small decrease tendency. Except for TH-IFR/4A/PP1 FR material, all the other FR PP composites show the similar mechanical properties. For the TH-IFR/4A/PP1 FR material with the best flame retardancy, its tensile strength and elongation at break achieve 28.9 MPa and 31.6%, respectively. The incorporation of 1 wt% 4A can slightly increase the tensile strength, but decrease the elongation at break to a certain degree. In total, this FR PP material possesses a good comprehensive performance.

## 4. Conclusions

In this study, a novel TH-IFR of ME PA·PER was successfully synthesized through hydrothermal reaction. The 4A was used as a synergist to further improve the flame retardancy of the prepared FR PP composite. The results of LOI, UL-94, CCT, TGA, FESEM-EDS, FT-IR and Raman measurements demonstrate that the incorporation of only 1 wt% 4A synergists can achieve the optimum synergistically flame-retardant effect. The corresponding TH-IFR/4A/PP1 FR material possesses the enhanced LOI of 30.1% and the vertical burning rating of UL-94 V-0. In addition, relative to pure PP, the cone calorimeter parameters of pHRR, THR, pSPR, pMLR and av-EHC of the TH-IFR/4A/PP1 FR compounds are also significantly decreased. The involved synergistically flame-retarding mechanism of 4A can be explained by the significantly improved quality and quantity of the solid-phase charred layers, which is realized through formation of SiO_2_ and Al_2_O_3_ particles, and also participation of PP macromolecular chains in the final char layer formation during burning. Furthermore, the improved dispersion and compatibility of TH-IFR fillers in the PP substrate due to the adsorption of 4A is beneficial to the improvement of flame retardancy. It is worth noting that the incorporation of 1 wt% 4A particles also helps to improve the mechanical properties (mainly the tensile strength) of the FR PP composite to a certain degree. With this good comprehensive performance, the prepared FR PP materials show the broader application prospects in the field of fire-proof requirements.

## Figures and Tables

**Table 1:** Formulation and flammability of pure PP and PP composites.

Sample	Composition (%)			Flammability		
	PP	TH-IFR	4A	LOI (%)	UL-94	Drpping
PP	100.0	-	-	19.0 (±0.6)	No rating	Yes
TH-IFR/PP	78.0	22.0	-	27.6 (±0.7)	V-1	No
TH-IFR/4A/PP1	78.0	21.0	1.0	30.1 (±0.3)	V-0	No
TH-IFR/4A/PP2	78.0	20.0	2.0	28.5 (±0.6)	V-1	No
TH-IFR/4A/PP3	78.0	19.0	3.0	27.0 (±0.5)	V-2	Yes
TH-IFR/4A/PP4	78.0	18.0	4.0	25.9 (±0.4)	No rating	Yes

**Table 2:** The CCT parameters of pure PP, TH-IFR/PP, TH-IFR/4A/PP1, TH-IFR/4A/PP2 and TH-IFR/4A/PP4 FR material.

Sample	TTI (s)	pHRR (kW/m^2^)	THR (MJ/m^2^)	pSPR (m^2^/s)	pMLR (g/s)	av-EHC (MJ/kg)	FPI (s·m^2^/kW)	Residue (%)
PP	28	850.19	108.52	0.129	0.181	40.44	0.033	0
TH-IFR/PP	21	390.28	99.16	0.066	0.097	32.82	0.054	5.64
TH-IFR/4A/PP1	21	212.42	90.04	0.045	0.056	30.26	0.099	9.08
TH-IFR/4A/PP2	22	318.76	97.20	0.054	0.096	32.66	0.069	6.38
TH-IFR/4A/PP4	23	474.54	101.34	0.074	0.115	33.80	0.048	2.21

**Table 3:** The TGA parameters of pure PP and FR PP composites.

Sample	T_onset_ (°C)	T_max_ (°C)	MDR (%/min)	C_700 °C_ (%)	T_700 °C_ (%)	Variation (%)
PP	400	449	2.61	0	-	-
TH-IFR/PP	302	446	2.50	9.61	8.42	14.13
TH-IFR/4A/PP1	290	448	2.16	12.75	8.86	43.39
TH-IFR/4A/PP2	303	446	2.42	10.67	9.29	14.85
TH-IFR/4A/PP3	314	447	2.53	10.34	9.73	6.27
TH-IFR/4A/PP4	333	446	2.61	9.72	10.16	−4.33

**Table 4:** The DSC parameters of pure PP and FR PP composites.

Sample	T_c_ (°C)	ΔH_c_ (J/g)	T_m_ (°C)	ΔH_m_ (J/g)	X_c_ (%)
PP	114.55	116.2	166.54	99.50	47.61
TH-IFR/PP	122.99	77.42	166.80	61.71	37.85
TH-IFR/4A/PP1	120.80	82.40	167.32	74.27	45.56
TH-IFR/4A/PP2	121.16	89.51	166.75	78.47	48.14
TH-IFR/4A/PP3	122.49	89.90	166.19	79.49	48.76
TH-IFR/4A/PP4	122.75	90.27	165.49	82.99	50.91

**Table 5:** The mechanical properties of pure PP and FR PP composites.

Sample	Tensile Strength (MPa)	Elongation at Break (%)
PP	30.3 (±0.3)	77.5 (±2.1)
TH-IFR/PP	27.6 (±0.4)	47.7 (±1.0)
TH-IFR/4A/PP1	28.9 (±0.2)	31.6 (±1.8)
TH-IFR/4A/PP2	27.8 (±0.1)	43.3 (±2.8)
TH-IFR/4A/PP3	27.6 (±0.6)	44.0 (±3.0)
TH-IFR/4A/PP4	27.5 (±0.2)	44.6 (±2.9)

## Data Availability

Not applicable.

## Supplementary Materials

The following supporting information can be downloaded at: https://www.mdpi.com/article/10.3390/polym15020374/s1, Figure S1: Digital photo of the sample pellets and injected samples; Figure S2: FT-IR spectra of PA, PER and intermediate PA⋅⋅⋅PER⋅⋅⋅PA; Figure S3: Digital photos of carbon residue for TH-IFR/4A/PP4; Figure S4: The stress-strain curves of pure PP and FR PP composites; Table S1: The element content of TH-IFR and 4A estimated from EDS surveys; Table S2: The element content of carbon residue for TH-IFR/PP and TH-IFR/4A/PP1 estimated from EDS surveys.
