# Supplementary material for: Synergistic Effect of 4A Molecular Sieve on Intumescent Ternary H-Bonded Complex in Flame-Retarding of Polypropylene
Source: Polymers (Basel). 2023 Jan 10;15(2):374. doi: 10.3390/polym15020374 (PMC9861832; doi:10.3390/polym15020374)
Supplement: Supplementary file 1 [file polymers-15-00374-s001.zip › polymers-2042535-supplementary.pdf]

Supporting Information

Synergistic Effect of 4A Molecular Sieve on Intumescent Ternary H-bonded Complex in Flame-Retarding of Polypropylene

Qilin Wen, Yinghong Chen*, Xin Wang, Haoran Pei

State Key Laboratory of Polymer Materials Engineering, Polymer Research Institute of Sichuan University, No. 24 South Section 1, Yihuan Road, Chengdu 610065, China

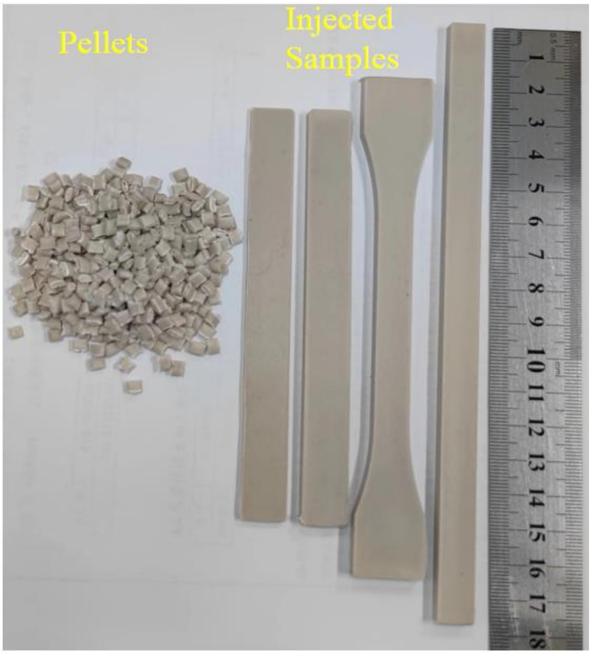

Figure S1. Digital photo of the sample pellets and injected samples.

* Corresponding author. *E-mail address:* johnchen@scu.edu.cn

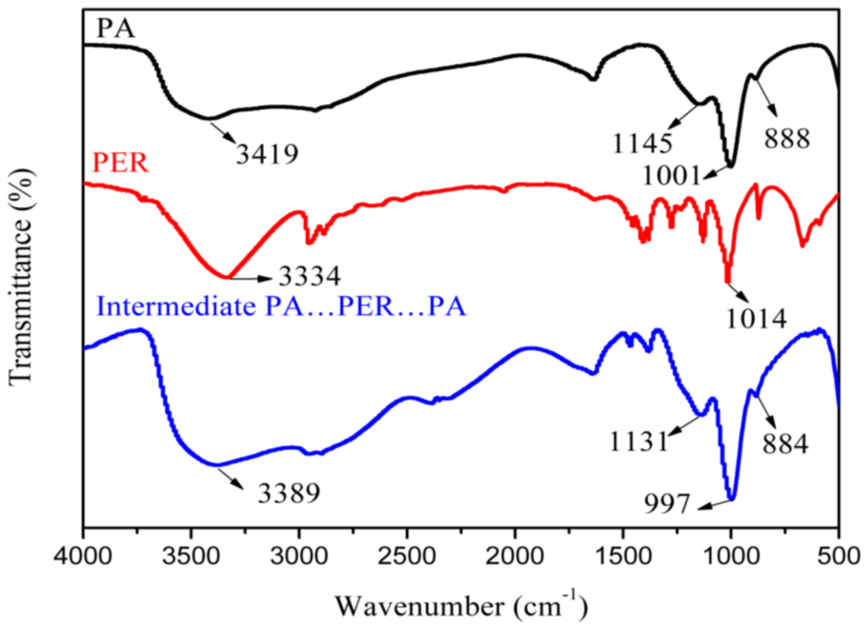

Figure S2. FT-IR spectra of PA, PER and intermediate PA···PER···PA.

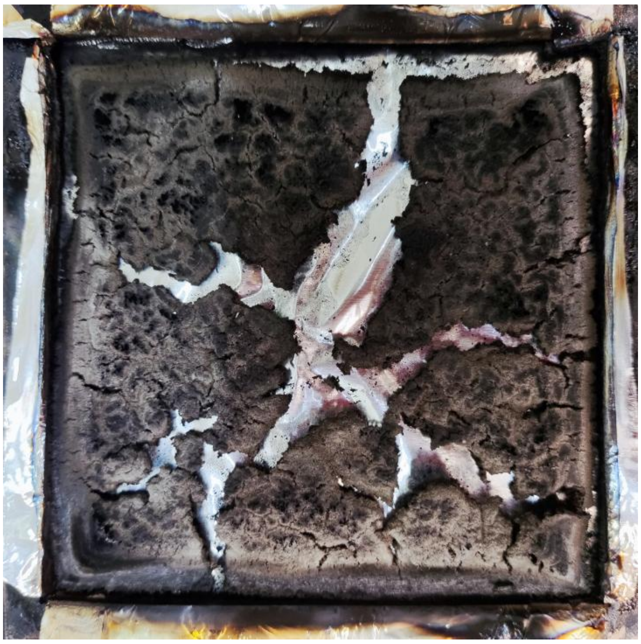

Figure S3. Digital photos of carbon residue for TH-IFR/4A/PP4.

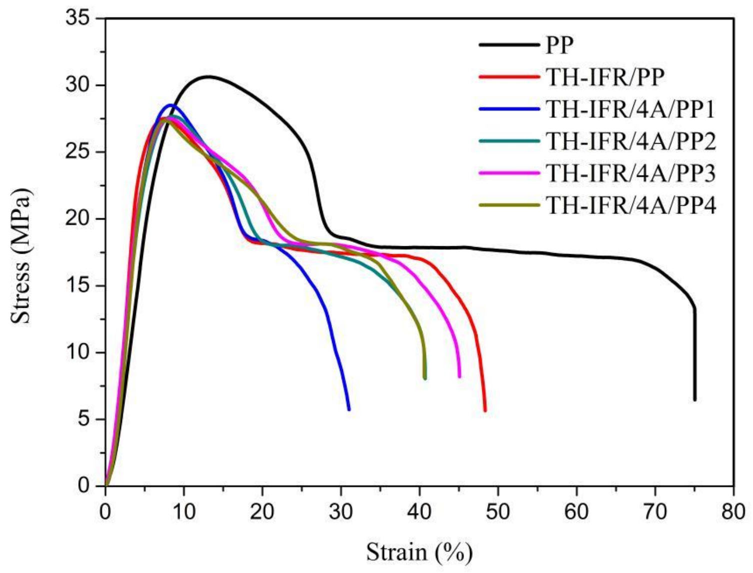

Figure S4. The stress-strain curves of pure PP and FR PP composites.

Table S1 The element content of TH-IFR and 4A estimated from EDS surveys

Sample	C (at%)	N (at%)	O (at%)	P (at%)	Na (at%)	Al (at%)	Si (at%)
TH-IFR	36.26	30.47	26.73	6.54	-	-	-
4A	-	-	49.83	-	13.41	18.02	18.74

Table S2 The element content of carbon residue for TH-IFR/PP and TH-IFR/4A/PP1 estimated from EDS surveys

Sample	C (at%)	N (at%)	O (at%)	P (at%)	Al (at%)	Si (at%)
TH-IFR	40.65	3.31	35.17	20.87	-	-
4A	53.68	1.45	29.71	12.87	1.20	1.09
